# Cigarette Smoking and Its Financial Burden among Iranian Households: Evidence from Household Income and Expenditures Survey

**DOI:** 10.34172/jrhs.2020.28

**Published:** 2020-10-13

**Authors:** Enayatollah Homaie Rad, Mohammad Hajizadeh, Satar Rezaei, Anita Reihanian

**Affiliations:** ^1^Social Determinants of Health Research Center, Guilan University of Medical Sciences, Rasht, Iran; ^2^School of Health Administration, Dalhousie University, Halifax, Canada; ^3^Research Center for Environmental Determinants of Health, Health Institute, Kermanshah University of Medical Sciences, Kermanshah, Iran; ^4^Guilan Road Trauma Research Center, Guilan University of Medical Sciences, Rasht, Iran

**Keywords:** Cigarette smoking, Expenditures, Determinants, Socioeconomic status, Iran

## Abstract

**Background:** The financial burden of cigarette smoking on households’ budget is not well documented in Iran. We aimed to identify the determinants of cigarette consumption and its financial burden among households in Iran.

**Study design:** A cross-sectional study.

**Methods:** A total of 39,864 Iranian’s households from 31 provinces were included in the analysis. Data on sociodemographic and socioeconomic characteristics (age, sex, household size, education level, employment status, income and wealth index), living area, number of cigarettes smoked and cigarette expenditures for households were extracted from the 2016 Household Income and Expenditures Survey (HIES). Tobit model was used to identify the determinants of cigarette smoking frequency and expenditures among Iranian households.

**Results:** The average number of cigarettes smoked and cigarettes expenditures by all household members was 85.25 cigarettes and US$ 2.64 per month. Living in urban areas, wealth index of households, household income, household size and low educational attainment of household members were positively associated with frequency and expenditures of cigarette smoking. Results also indicated increasing patterns in the number of cigarettes smoked and cigarettes expenditures from east to west of the country. East Azerbaijan, Hamadan, Markazi and Chaharmahal va Bakhtiari provinces had higher cigarette smoking frequency and expenditures in Iran.

**Conclusions:** Tobacco control interventions in Iran should focus more on households living in urban areas and low-educated households. As the frequency of cigarette smoking was higher in the western region of Iran, comprehensive tobacco control policies should be adopted in western provinces.

## Introduction

Cigarette smoking is one of the most important preventable causes of cardiovascular diseases, cancer and respiratory tract infection, among others ^1^. The annual number of deaths due to smoking is much higher than the total deaths from AIDS, alcohol, addictive drugs, accidents, murder and suicide globally ^2^. Smoking has an important role on the global burden of diseases. It is the risk factor for many non-communicable diseases such as cancer ^3^. Smoking was the direct cause of 4,623 deaths in Iran in 2012^4^.

In addition to adverse health outcomes of smoking for the smokers, it is responsible for a significant financial healthcare burden for society due to smoking-attributable diseases^5^. Cigarette tax, banning the sale of cigarettes for younger age-groups and smoking ban on public places are some of the strategies to reduce tobacco consumption ^6^. Despite these tobacco control policies to reduce smoking prevalence, the monetary profits of smoking cigarettes for the manufacturing companies and excessive smuggling of this substance make cigarettes available cheaply in different places, especially in low- and middle-income countries (LMICs) ^7^.

Although tobacco provides significant tax revenues to governments, smoking has a significant impact on households’ budgets as well as the health status of individuals and societies as a whole ^8^. Tobacco accounts for 6% of all healthcare costs worldwide. The total economic cost of smoking was US$1,436 billion in 2012, equivalent to 1.8% of gross domestic products (GDP) of the entire world ^9^. The total economic cost of smoking-attributable diseases in Iran was estimated to be US$1.46 billion accounting for approximately 0.26% of Iran’s GDP in 2014 ^10^.

Studies have shown that tobacco and poverty constitute a defective cycle that exacerbates each other^8^. In most countries, smoking is more common among low-income communities, with 84% of smokers living in LMICs ^9^. Due to budget constraints, the money spent on cigarettes cannot be used for the consumption of basic needs such as nutrition, housing, education and health. Smoking can worsen poverty because smokers and their families are more susceptible to early death from a heart attack, cancer, respiratory distress and other smoking-attributable diseases. As poor households often do not have enough protection against healthcare spending, smoking-attributable diseases can result in catastrophic out-of-pocket payment for healthcare and lead to impoverishment.

According to WHO, a reduction was in the number of smokers by 20 million in the world between 2015 and 2025 ^11^. While the reduction in the number of smokers among women is satisfactory to reach the 2025 estimate, slower progress among men is a concern ^12^. Thus, it is essential to identify factors affecting consumption of smoking to provide up-to-date information for formulating a tobacco control policy worldwide. Although there have been several studies^13-15^ that aimed to assess the determinants of smoking in different countries, determinants of cigarette smoking are rarely documented in Iran as a whole. The existing studies highlighted the importance of personal, behavioural, environmental, and social factors on smoking in Iran. Nevertheless, the financial burden of smoking in Iran has not been investigated in Iran.

 We aims to fill this gap in the literature by investigating the determinants of cigarette smoking frequency and expenditures among Iranian households.

## Methods

 In this cross-sectional observational study, 39,864 households from 31 provinces of Iran were enrolled. The required data were extracted from the 2016 Household Income and Expenditures Survey (HIES), conducted by the Iranian Statistical Center (ISC). In this survey, data on sociodemographic and socioeconomic characteristics (age, sex, household size, education level, employment status, income and wealth index), living area, number of cigarettes smoked and cigarette expenditure for households were obtained from the HIES.

 Data were collected using face-to-face interviews with the household head. The HIES collects information from all households living in rural and urban areas of Iran, excluding temporary foreign residents. The survey uses a standard questionnaire, designed under supervisions and recommendations of the United Nations. Households were chosen using a clustered random sampling technique and counties as clusters into urban and rural regions.

###  Study variables

The two outcome variables of interest in the study were the number of cigarettes smoked per month and monthly expenditures on cigarettes in the household. Cigarette smoking is defined as smoking of regular cigarette brands and does not contain hookahs and e-cigarettes. Based on the previous studies ^3,16,17,18^ annual income, place of residence (urban or rural region), wealth index, number of females in each household, household size, number of illiterate people in each household, number of people with a university degree in each household, number of household members between 17 and 30 yr old were included in the analysis as explanatory variables in the analysis. We used the modified principal component analysis (MPCA) to calculate the wealth index of the household. Type of house ownership, home area surface (less than 65 m^2^, between 65 and 110 m^2^, between 110 and 200 m^2^, more than 200 m^2^), type of house skeleton, type of major building materials, ownership of cars, bicycles, motorcycles, radios, televisions, videos, computers, mobile phone, refrigerator, stove, vacuum machines, air conditioners, drinking waters, electricity, gas, central heating and cooling systems were entered in the MPCA. Based on wealth scores obtained from the MPCA, households were divided into ten wealth decile groups.

###  Statistical analysis 

 Due to zero-inflated data of our outcome variables, we used the following Tobit regression model to examine the association between the number of cigarette smoking (or cigarette expenditures) in the household and its main determinants:

 yit=β1+ β2inci+β2inc2i+β4wealthi+ β5femalei+ β6agei+β7iliti+β8hhsi+β9unii+β10worki+ Ei, (1)

 where y is the outcome variable of interest, inc is the yearly income of the households (US$), inc2 indicates the second-order of households’ annual income, wealth denotes wealth status of the household, female is the number of females in the household, age indexes the number of household members aged between 17 and 30 years old, hsize is the household size and ilit is the percentage of illiterate members of the household. uni is the percentage of household members with a university degree. work denotes the percentage of people working in the household. The population weighting method was used to pool the data collected from the urban and rural regions. To have a better understanding of differences in cigarette consumption and spending across different regions of Iran, we also visually illustrated the spatial distribution of the number cigarettes smoked per month and monthly expenditures on cigarettes in the household in all counties in Iran. All statistical analyses were performed at 0.05 significance levels. All analyses were conducted using STATA software SE v 13.1.

## Results

 Each Iranian household smoked 85.25 cigarettes per month and spent US$ 2.64 on cigarettes per month. The average yearly income of Iranian households was US$ 4,847, and the average household size was 3.55. Besides, 52.13% of household members were women, and 30.13% were illiterate, 26.7% employed and 14.2% had a university degree. Out of 39,864 households, 33015 (82.81%) households did not smoke any cigarettes over the period of study.

Table 1 shows the results of the Tobit regression for the determinants of the number of cigarette smoking and cigarette expenditures. As reported in the table, the results of the Variance Inflation Factor (VIF) test did not find high collinearly between explanatory variables. The coefficient on living in urban areas was 3.87 for the number of cigarettes smoked model and 142.02 for cigarette expenditures model. Both these coefficients are statistically significant; (P=0.001 of both coefficients) indicating that people living in urban areas, on average, smoked more cigarettes and spent more on cigarettes. The higher percentage of household members with an academic degree was negatively associated with both cigarette consumption and its spending. In contrast, there were positive and significant associations between the percentage of the illiterate member of household and household’s cigarette consumption and expenditures (P=0.001 for both coefficients). There was a positive and significant relationship between the number of household members aged 17 and 30 years old and cigarette expenditures (P=0.018). The results also suggested significant and negative relationships between the number of females in the household and cigarette expenditures and cigarette consumption (P=0.001 for both coefficients). Furthermore, the wealth of household had a positive impact on the number of cigarettes smoked and cigarette expenditures. After controlling for other covariates, the number of cigarettes smoked (cigarette expenditures) in the household increased until 7^th^ (6^th^) decile, before decreasing in higher wealth deciles. The coefficients on the first and second orders of household income indicated that the relationships between income and cigarette consumption and spending is nonlinear and had a peak (see the first order of income had a significant positive relationship with dependent variables while the coefficient of the second-order of income was significant and negative).

**Table 1:** Tobit regression results for the determinants of the number of cigarette smoking and expenditures on cigarettes among Iranian households

**Variable**	**Expenditures on cigarettes**			**Number of cigarette smoking**		
	**Coefficient**	**SE**	***P*** ** value**	**Coefficient**	**SE**	***P*** ** value**
Household size	2.71	0.18	0.001	86.84	6.28	0.001
Number of household members aged 17 -30 years old	0.59	0.25	0.018	10.26	8.40	0.222
Number of females in the household	-0.76	0.12	0.001	-12.38	3.34	0.001
Urban	3.87	0.42	0.001	142.02	14.35	0.001
Number of household members working	0.06	0.22	0.782	4.44	7.60	0.558
Percentage of household members with a university degree	-4.41	0.29	0.001	-144.89	10.02	0.001
Percentage of illiterate members in the household	2.26	0.24	0.001	75.48	8.35	0.001
Wealth deciles						
2	2.89	1.08	0.008	124.90	37.41	0.001
3	4.55	1.07	0.001	186.51	37.14	0.001
4	6.24	1.07	0.001	273.76	36.93	0.001
5	6.67	1.08	0.001	275.92	37.21	0.001
6	7.02	1.09	0.001	302.36	37.41	0.001
7	7.15	1.10	0.001	298.62	37.79	0.001
8	4.48	1.12	0.001	207.74	38.66	0.001
9	3.57	1.14	0.002	190.97	39.38	0.001
10	1.81	1.20	0.134	128.96	41.39	0.002
Annual income (1000 dollars)	1.05	0.11	0.001	28.55	3.99	0.001
Annual income (1000 dollars) squared	-0.02	0.00	0.001	-0.58	0.14	0.001
Consent	-41.05	1.14	0.001	-1421.48	39.70	0.001
VIF test results	2.238	2.238

**Figure 1:**
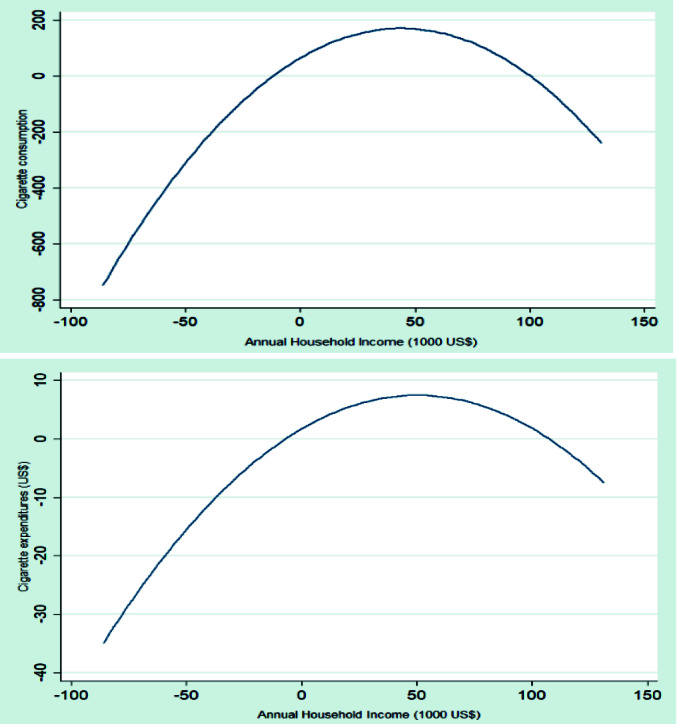
Fit plots of the relations between household income and household number of cigarettes smoked and spending on cigarettes per month in Iran

Figure 1 shows the relationship between household income and household number of cigarettes smoked and cigarette expenditures in fit plots. Accordingly, there was a peak for at the annual income of US$ 30,000 and US$ 50,000 for the number of cigarettes smoked and cigarette expenditures, respectively.

**Figure 2:**
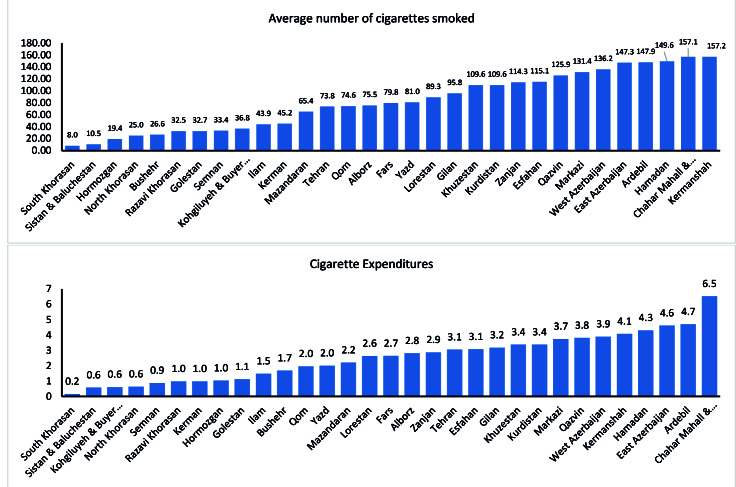
Household average number of cigarettes smoked and cigarette expenditures (US$) per month among Iranian provinces

Figure 2 shows the average household number of cigarettes smoked and cigarette expenditures among Iranian provinces (after adjusting for household size). Households living in South Khorasan, Sistan va Baluchestan, Kahkilooye va Boirahmad with US$ 0.19, US$ 0.46 and US$ 0.53, respectively, had the lowest monthly expenditures on cigarettes. Charaharmahal va Bakhtiari, East Azerbaijan and Ardebil with US$ 6.15, US$4.84 and US$4.64, respectively, had the highest household expenditures on cigarettes. The lowest average numbers of cigarette smoke in the household were found in Sistan & Baluchestan (8.33), South Khorasan (9.75) and Hormozgan (16.44). East Azerbaijan (175.4), Hamedan (155.31), Markazi (148.75) and Charaharmahal va Bakhtiari (148.18) had the highest numbers of cigarette smoked in the household.

**Figure 3:**
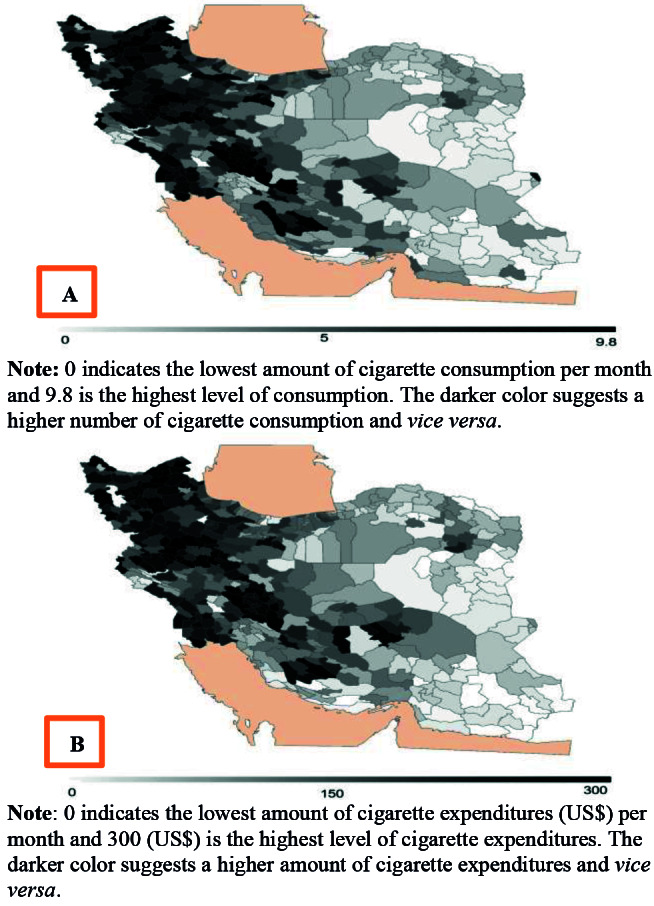
The average number of cigarettes smoked (A) and cigarette expenditures in US$ (B) per month among Iranian counties

Figure 3 shows the consumption and expenditures of cigarette by Iranian counties. The numbers of cigarette smoked, and expenditures on cigarettes were shown in the spectrum below the maps. The counties with higher amount of expenditures (consumption) are darker than others. The western compared to the eastern region in Iran generally are darker than other regions; indicating the higher cigarette expenditures (consumption) in the western counties compared to eastern counties in Iran.

## Discussion

We aimed to find the determinants of the consumption and spending on cigarettes smoking among Iranian households. We found that Iranian households, on average, smoked 85.25 cigarettes and spent US$ 2.64 on cigarette smoking per month. The results indicate that residing in urban areas had a positive and significant relationship with smoking and spending on cigarettes in Iran. There was no relationship between the number of people employed in the household and the number of smoking cigarettes in Iranian households. These results are in contrast with another study that showed a positive association between the unemployment rate and cigarette consumption in Iran.^15^ Unemployment increases smoking expenditures in Iran^19^. It also increases psychosocial disorders like emotional isolation or inability to control and these factors are important drivers of smoking in people ^20^.

Our study showed that higher educational attainments (higher percentage of members with a university degree in the household) in the household had a negative effect on the consumption and spending on cigarettes among Iranian households. In contrast, lower educational attainments (i.e., a higher percentage of illiterate members in the household) had a positive relationship with tobacco consumption. A similar relationship between literacy rate and smoking too ^21^. For instance, increasing illiteracy rates at the household level would increase smoking ^19^. Another study concluded that increasing literacy rate decreases smoking. It is obvious that people with higher education levels have more awareness about the harms of smoking so they smoke less than others ^15^.

A study in Isfahan, Iran also showed that lower education attainment of fathers in the household increased the probability of smoking in both genders, especially among girls in households ^22^. The results also indicated that the number of household members aged 17 -30 had a positive relationship with household spending on cigarette expenditures.

The results of our findings indicated that households with middle socioeconomic status (as measured by wealth index) were more likely to smoke and spend on cigarettes in Iran. Specifically, the relationships between income and cigarette consumption and expenditures increased to an annual household salary of US$ 2,898 and then decreases subsequently. This is because two contradictory effects of plausible mediators in the relationship between wealth and smoking. First the income effect: Low income people have lower ability to buy cigarette, so they buy less cigarette than the rich. Second, the awareness effect: Higher income groups are more educated ones and know the harms of smoking, so they smoke less than the poor. These two contradictory mediators led to a nonlinear relationship between income (wealth) and smoking ^23,24^. Dortaj found a significant negative relationship between the monthly income with drug abuse^25^. The association between income and smoking was not linear^15^. In other words, although smoking rate increased with income, the consumption of smoking started to decline after a certain level of income.

We found a wide variation in the number of cigarettes smoked and cigarette expenditures among Iranian provinces. The provinces of Sistan & Baluchestan, South Khorasan and Hormozgan had the lowest cigarette consumption whereas the provinces of East Azerbaijan, Hamedan, Central and Chaharmahal va Bakhtiari had the highest consumption. A study by Bakhshani also showed higher smoking consumption in Ardebil and ChaharMahal va Bakhtiari, and lower smoking consumption in provinces such as Bushehr and Sistan va Baluchestan ^26^. While South Khorasan, Sistan va Baluchestan and Kohkiluyeh va Boyerahmad provinces had the lowest monthly average household expenditure on cigarette smoking, provinces of ChaharMahal va Bakhtiari, East Azerbaijan and Ardebil had the highest average monthly spending on cigarette smoking. The order of the provinces based on number of cigarette smoking per month does not correspond to their order based on the cigarette expenditures. Although this inconsistency may be due to self-reporting bias across different provinces of Iran, it can also be explained by purchasing cheaper brands in some provinces and more expensive ones in others.

The results of this study suggested higher smoking consumption and spending on cigarette smoking in Azerbaijani provinces of Iran compared to other provinces. The Iranian Azerbaijan region includes the north-west and part of the central and southern parts of Iran. As indicated in the map, cigarette smoking consumption and cigarette expenditure in these regions are higher compared to other regions in Iran. The eastern and southeastern provinces of the country had much less cigarette smoking than other provinces. This may be attributed to the use of other alternatives tobacco products which could be a substitute for cigarette smoking ^27,28^ For example, Nass is one of the most favorite drugs which is used instead of cigarette^29^ or Waterpipes ^30^.

 This study had some limitations. First, individual-level information was not available in HIES; thus, we used household-level data to the determinants of cigarette consumption and its financial burden among households in Iran. Second, due to unavailability of information, we could not assess the impact of other factors such as societal norms environmental factors (advertising) and cultural factors (traditional uses of tobacco, acculturation) on cigarette consumption and spending on cigarette among households. Third, the self-reported data were prone to recall bias. Under-reporting bias might also be inevitable.

## Conclusion

 Iranian household, on average, smoked 85.25 cigarettes and spent US$2.64 on cigarette consumption in 2016. Smoking is a significant part of the overall spending among Iranian households. In addition to the cost of cigarettes consumption, smoking also drives substantial direct healthcare spending on treatment of smokers and people exposed to smoke in Iran. The number of males in the household associated with higher cigarette consumption and expenditures. The latter finding could be due to social, cultural, religious and demographic factors, and also the fact that smoking in women compared to men is considered inappropriate in Iran. Due to higher cigarette consumption in urban areas and low-educated households, tobacco control interventions in Iran should focus more on households living in urban areas and less-educated households. There exists a wide variation in the number of cigarettes smoked and cigarette expenditures among Iranian provinces. As the frequency of cigarette smoking was found to be higher in the western region of Iran, comprehensive tobacco control policies should be adopted in western provinces.

## Acknowledgements

 This study was confirmed ethically by Deputy of Research, Guilan University of Medical Sciences. Ethics code: IR.GUMS.REC.1397.488.

## Conflict of interest

 Authors declared no conflict of interests.

## Funding

 This study was supported by Social Determinants of Health Research Center, Guilan University of Medical Sciences.

## Highlights

- Households with middle socioeconomic status are more likely to smoke and spend on cigarettes in Iran.
- The relationships between income and cigarette consumption and expenditures are nonlinear.
- The eastern and southeastern provinces of the country had much less cigarette smoking than other provinces.
- Smoking consumption and spending on cigarette smoking was higher in Azerbaijani provinces of Iran compared to other provinces.
